# Prospective Analysis of Squamous Cell Carcinoma Antigen-1 and -2 for Diagnosing Sinonasal Inverted Papilloma

**DOI:** 10.3390/jcm13092721

**Published:** 2024-05-06

**Authors:** Hitoshi Hirakawa, Taro Ikegami, Masatomo Toyama, Yurika Ooshiro, Tomoyo Higa, Hidetoshi Kinjyo, Shunsuke Kondo, Norimoto Kise, Yukashi Yamashita, Mikio Suzuki

**Affiliations:** Department of Otorhinolaryngology, Head and Neck Surgery, Graduate School of Medicine, University of the Ryukyus, 207 Uehara, Nishihara-cho, Nakagami-gun, Okinawa 903-0215, Japan; aoi23@med.u-ryukyu.ac.jp (H.H.); ikegami@med.u-ryukyu.ac.jp (T.I.); puyoraer99110@gmail.com (M.T.); yurikaaa.0108@gmail.com (Y.O.); tomoyo_12_26@hotmail.co.jp (T.H.); hidechanman223@yahoo.co.jp (H.K.); kouhouiinn@yahoo.co.jp (S.K.); norimoto7@gmail.com (N.K.); y.yukashi@gmail.com (Y.Y.)

**Keywords:** inverted papilloma, squamous cell carcinoma antigen 1, squamous cell carcinoma antigen 2, differential diagnosis

## Abstract

**Background**: The goal of this research was to confirm whether preoperative serum squamous cell carcinoma antigen (SCCA)-1 and -2 levels are useful diagnostic markers for sinonasal inverted papilloma (IP) in a prospective study. **Methods**: Participants were 102 patients who underwent consecutive endoscopic sinus surgery: 18 with IP, two with other types of papilloma, 77 with chronic rhinosinusitis, four with sinonasal cancer, and one with hemangioma. SCCA-1 and SCCA-2 were measured preoperatively by an automatic chemiluminescence immunoassay and an enzyme-linked immunosorbent assay, respectively. **Results**: SCCA-1 and SCCA-2 values were significantly correlated (r = 0.603, *p* < 0.001). Receiver operating characteristic analysis for differentiating papilloma (IP and other types of papilloma) from other diseases yielded an area under the curve of 0.860, with a Youden index of 1.75. Combined with SCCA-2 analysis, the detection system had a sensitivity and specificity of 0.65 and 0.98, respectively. While our study did not find a strong link between SCCA levels and skin or lung diseases, smoking status may influence SCCA levels in IP patients (*p* = 0.035). We recommend a cutoff value of 1.8 ng/mL for SCCA-1 in IP diagnosis. **Conclusions**: SCCA-1 and SCCA-2 when combined with imaging and pathology hold promise for enhancing the preoperative detection of IP, which would be a valuable contribution to clinical practice.

## 1. Introduction

Squamous cell carcinoma antigen (SCCA) in serum is commonly used as a tumor marker for squamous cell carcinoma (SCC) in uterine cervical and head and neck cancers [1]. Molecular biology research has established that SCCA is produced by two homologous genes, namely, *SCCA-1* and *SCCA-2*. *SCCA-1* and *SCCA-2* are encoded by the serine protease inhibitor family (SERPINB3/B4) and are located within the serpin cluster on chromosome 18q21.3 [2]. *SCCA-1* encoded by SERPINB3 inhibits lysosomal cathepsins, while *SCCA-2* encoded by SERPINB4 inhibits cathepsin G [3]. SCCA-1 regulates cell death associated with protease activation [4,5]. SCCA is distributed in normal squamous epithelium and epithelial tissues. Notably, elevated levels of SCCA have been identified in benign lung and skin lesions [6,7], as well as uterine cervical and head and neck cancers. Our previous study found that a high *SCCA-2*/*SCCA-1* mRNA ratio is a significant predictor of disease recurrence in patients with SCC of the head and neck without a human papillomavirus infection [8]. Another study found that pretreatment serum SCCA levels can indicate tumor stage and stromal invasion in cervical cancer [9].

Inverted papilloma (IP) in the paranasal sinus is a benign tumor but is clinically significant due to its aggressive features. In particular, it tends to recur and may even undergo malignant transformation. Recent studies have suggested that epidermal growth factor receptor mutation might be instrumental in the malignant transformation of IP [10]. Current meta-analyses estimate that the recurrence rate after endoscopic resection of IP is around 12%, while the rate of malignant transformation is around 10% [11,12,13]. Given that the recommended treatment for IP is complete surgical removal, precise preoperative diagnosis and assessment of IP spread are crucial. Recurrent cases present anatomical changes due to prior surgery, such as bony overgrowth and significant scarring. Also, IP often mimics symptoms of unilateral chronic rhinosinusitis (CRS) with polyps due to blockage of the sinus ostium [14]. Surgical methods differ significantly between IP and CRS. Hence, precise preoperative diagnosis and evaluation of IP extent are vital.

Yasumatsu et al. identified elevated serum SCCA levels in patients with IP in the sinonasal tract [15]. In our previous study, we found that 81.8% of patients with IP and 90.3% of those with recurrent IP had significantly higher SCCA levels than patients with inflammatory conditions [16]. Notably, both *SCCA-1* and *SCCA-2* mRNA expression values were markedly elevated in IP patients compared with the inflammatory group [16]. Several studies subsequently reported similar results [17,18,19]. However, because all of these studies were retrospective case series with selection bias, we sought to confirm the significance of SCCA values for the preoperative detection of IP in a prospective study. In addition, smoking has been reported to affect SCCA values in IP and CRS, so smoking status should be considered when evaluating the significance of SCCA [20]. A recent study indicated that SCCA combined with cytokeratin fragment antigen 21-1 differentiated IP from CRS with high sensitivity and specificity [21].

It has become possible to detect SCCA-1 and SCCA-2 separately [22], with SCCA-2 known to be a biomarker for atopic dermatitis [23]. We aimed to confirm whether serum SCCA-1 and-2 levels are useful diagnostic markers during preoperative evaluation of patients with IP in a prospective study.

## 2. Materials and Methods

### 2.1. Patients

The participants in this prospective cohort study were 106 consecutive patients who underwent endoscopic sinus surgery for sinonasal lesions from October 2018 to February 2020. All patients provided written informed consent to participate. The surgeries were performed by 4 surgeons. The exclusion criteria were concurrent malignant lesions, except for sinonasal lesions, and receiving anti-cancer therapy for malignant lesions.

The study protocol was approved by the Institutional Review Board of the University of the Ryukyus (protocol number H23.6-7). This study was conducted in accordance with the ethical principles set forth in the 1975 Declaration of Helsinki, as revised in 2008.

### 2.2. SCCA Measurement

Serum SCCA levels were measured before and 1–3 weeks after surgeries. The surgical specimens were subjected to pathological examination. Purified recombinant human SCCA-1 and SCCA-2, which were expressed in HEK293T cells and purified by immobilized metal ion affinity chromatography, were purchased from RayBiotech, Inc. (catalog number 230-30005 and 230-30014, respectively; Peachtree Corners, GA, USA). Serially diluted SCCA-1 and SCCA-2 were measured using an automatic chemiluminescence immunoassay (Architect, Abbott Laboratories, Wiesbaden, Germany) [24,25] to check their cross-reactivity at concentrations from 0 to 200 ng/mL. The cross-reactivity data are available in the Supplementary Materials. SCCA-1 can be measured without cross-reactivity to SCCA-2 in the Architect immunoassay. SCCA-2 was measured using an enzyme-linked immunosorbent assay (ELISA, SHINO-TEST Co., Tokyo, Japan), which is not affected by cross-reactivity with SCCA-1 [22].

### 2.3. Relationship between Clinical Characteristics and SCCA

The clinical features of IP include the Krouse classification [26], history of prior surgery, recurrence after surgery, and concomitant malignant lesions. Because patients with pulmonary and skin diseases also show elevated serum SCCA levels [6,7], these diseases and smoking and drinking habits were also recorded before surgery.

### 2.4. Statistical Analysis

Receiver operating characteristic (ROC) curve analysis was performed to differentiate IP and other types of papilloma from sinonasal lesions. The paired-sample Wilcoxon signed-rank test was used to analyze differences in preoperative and postoperative SCCA levels between patients with IP and those with CRS. All analyses were performed using SPSS for Windows, version 28.0 (SPSS Inc., Chicago, IL, USA). Statistical significance was set at *p* < 0.05. The above data are available in the Supplementary Materials.

## 3. Results

### 3.1. Patient Characteristics

**Figure 1:**
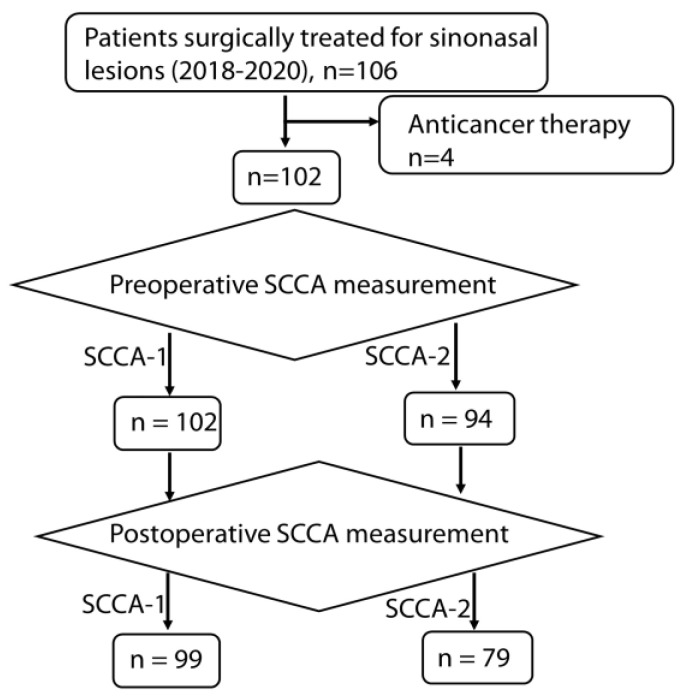
Participants and squamous cell carcinoma antigen (SCCA) measurements.

After excluding four patients based on the exclusion criteria, 102 remained for analysis (Figure 1). There were eighteen patients with IP, two with other types of papilloma (columnar cell papilloma and exophytic papilloma), four with sinonasal cancer (SCC, adenosquamous carcinoma, adenoid cystic carcinoma, and malignant melanoma), one benign tumor (hemangioma), and seventy-seven with CRS. CRS included eosinophilic CRS, fungal sinusitis, dental sinusitis, and sinus mucocele. The clinical characteristics of the patients are provided in Table S1 (Supplementary Materials).

The flow of patients in this study is shown in Figure 1. Although SCCA-1 was measured preoperatively in all 102 included patients, it was not measured postoperatively in three cases (malignant melanoma, one; chronic inflammation, two). SCCA-2 was measured in 94 patients preoperatively (92%) and in 79 postoperatively (77%) due to limited blood samples. The eight patients whose SCCA-2 was not measured preoperatively included one with malignant melanoma and seven with CRS. Patients with SCCA-2 measurements both preoperatively and postoperatively included thirteen with IP, sixty-two with chronic inflammation, two with sinonasal cancer, and one with hemangioma.

According to the Krouse classification, one IP case was stage I, four were stage II, thirteen were stage III, and zero were stage IV [26]. In the IP group, one patient had bilateral IP lesions, both were stage III, and six patients had previously undergone sinus surgery at other hospitals.

Two of the eighteen IP patients experienced recurrent lesions 6 months after surgery. At the initial visit to our hospital, one of these patients had extensive frontal sinus involvement and the other had bilateral sinus involvement. The bilateral case had incomplete resection due to intraoperative bleeding and underwent re-operation 3 years later, achieving remission for 1 year. In this patient, the preoperative SCCA-1 level was 6.0 ng/mL and the SCCA-2 level was 1.5 ng/mL at the first operation. Postoperatively, SCCA-1 and SCCA-2 levels were 5.3 ng/mL and 1.4 ng/mL, respectively. At the revision surgery, preoperative SCCA-1 and SCCA-2 were 9.9 ng/mL and 1.0 ng/mL, and postoperative SCCA-1 and SCCA-2 were 0.7 ng/mL and 0.5 ng/mL, respectively. After the second operation, both SCCA-1 and SCCA-2 remained below 1.0 ng/mL.

### 3.2. Relationship between Preoperative SCCA-1 and SCCA-2

**Figure 2:**
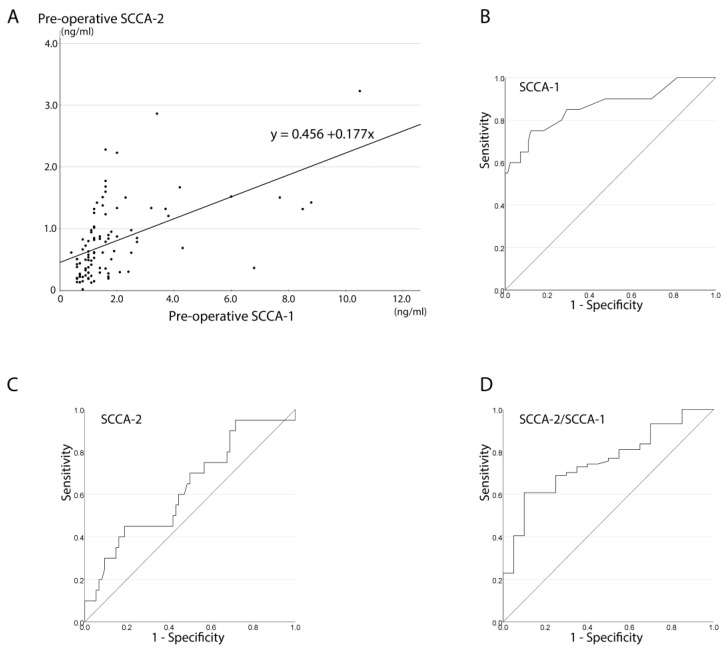
Relationship between squamous cell carcinoma antigen (SCCA)-1 and SCCA-2 and receiver operating characteristic (ROC) curves of SCCA-1, SCCA-2, and SCCA-2/SCCA-1 for discriminating inverted papilloma and other types of papilloma from other sinonasal diseases. (**A**) Relationship between preoperative SCCA-1 and SCCA-2 in all participants. (**B**) ROC analysis of preoperative SCCA-1 for differentiating papilloma lesions (inverted papilloma and other types of papilloma) from other sinonasal diseases (area under the curve [AUC], 0.863; Youden index, 1.75). (**C**) ROC analysis of preoperative SCCA-2 for differentiating papilloma lesions (inverted papilloma and other types of papilloma) from other sinonasal diseases (AUC, 0.629). (**D**) ROC analysis of preoperative SCCA-2/SCCA-1 for differentiating papilloma lesions (inverted papilloma and other types of papilloma) from other sinonasal diseases (AUC, 0.747).

The preoperative mean ± standard deviation (SD) in all patients was 1.8 ± 1.8 ng/mL for SCCA-1 and 0.8 ± 0.6 ng/mL for SCCA-2. Figure 2 shows the relationship between preoperative SCCA-1 and SCCA-2, which exhibited a significant correlation (r = 0.603, *p* < 0.001, Spearman’s rank correlation coefficient, Figure 2A). The linear model for SCCA-1 (*x*-axis) and SCCA-2 (*y*-axis) was y = 0.456 + 0.177x.

### 3.3. Preoperative SCCA-1 in Patients with Sinonasal Diseases

Table 1 shows preoperative SCCA-1 levels in patients with sinonasal disease. SCCA-1 levels were significantly higher in patients with IP (4.3 ± 3.0 ng/mL) than in those with CRS (1.2 ± 0.6 ng/mL, Mann–Whitney U test, *p* < 0.001). The case with columnar cell papilloma had SCCA-1 of 4.3 ng/mL, while the case with exophytic papilloma had SCCA-1 of 1.2 ng/mL. The SCCA-1 levels in patients with SCC and adenosquamous carcinoma were 1.4 and 1.6, respectively. ROC analysis for differentiating papilloma lesions (IP and other types of papilloma) from other diseases including CRS, sinonasal cancer, and benign nasal tumor showed an AUC of 0.863, with a Youden index of 1.75 (Figure 2B). Sensitivity and specificity were 0.75 and 0.88, respectively, when the SCCA-1 cutoff was set to 1.8 according to the Youden index. The positive predictive value (PPV) and negative predictive value (NPV) were 0.60 and 0.93, respectively.

### 3.4. Preoperative SCCA-2 in Patients with Sinonasal Diseases

Table 2 shows preoperative SCCA-2 levels in patients with sinonasal diseases. The values were higher in IP than in CRS, but the difference did not reach statistical significance (*p* = 0.056, Mann–Whitney U test). The mean values in patients with IP and those with CRS were 1.1 ng/mL and 0.7 ng/mL, respectively. SCCA-2 values showed no significant correlation with sinonasal diseases. ROC analysis for differentiating IP and other types of papilloma from other diseases based on preoperative SCCA-2 yielded an AUC of 0.629 (Figure 2C).

Preoperative SCCA-2/SCCA-1 ratios ranged from 0.03 to 1.54. Among the 94 patients in whom both SCCA-1 and SCCA-2 were measured preoperatively, 10 cases had an SCCA-2/SCCA-1 ratio of ≥1. These included one case with adenosquamous cell carcinoma and nine cases with CRS. The preoperative SCCA-2/SCCA-1 ratio was significantly lower in patients with IP than in those with other diseases (*p* < 0.001, Mann–Whitney U test; Table 1). Using the preoperative SCCA-2/SCCA-1 ratio, ROC analysis for differentiating IP and other types of papilloma from other diseases yielded an AUC of 0.755 (Figure 2D).

There were twenty-five patients who had SCCA-1 of ≥1.8 ng/mL (fourteen IP, one columnar cell papilloma, and ten CRS), as shown in Table 3. Of these 25 patients, all those with SCCA-1 of ≥3.4 ng/mL had IP or other types of papilloma.

**Figure 3:**
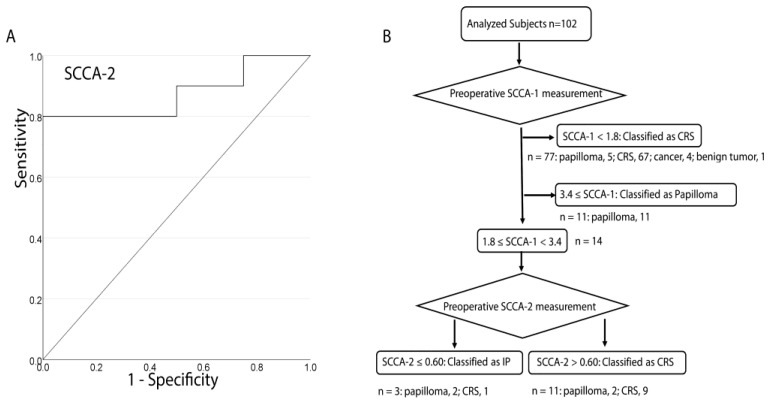
Protocol for discriminating inverted papilloma and other types of papilloma from other sinonasal diseases. (**A**) Receiver operating characteristic (ROC) analysis of preoperative squamous cell carcinoma antigen (SCCA)-2 to differentiate inverted papilloma and other types of papilloma from other sinonasal diseases in patients with SCCA-1 of ≥1.8 to <3.4 ng/mL (AUC, 0.875; Youden index, 0.56). (**B**) Diagnostic protocol using SCCA-1 and SCCA-2 based on the present study results.

For 14 patients with SCCA-1 from 1.8 to 3.4 ng/mL (Table S2, Supplementary Materials), ROC analysis for differentiating papilloma (IP and other types of papilloma) from CRS based on SCCA-2 yielded an AUC of 0.875, with a Youden index of 0.56 (Figure 3A). The IP status of patients with SCCA-1 of 1.8 to 3.4 ng/mL could be well differentiated depending on whether SCCA-2 was above or below 0.6 ng/mL. Thus, when SCCA-1 levels were less than 1.8, the likelihood of papilloma (IP and other types of papilloma) was only 6.5%. Conversely, when SCCA-1 levels ranged from 3.4 and above, the probability of papilloma diagnosis was 100%. For SCCA-1 values falling between 1.8 and 3.4, SCCA-2 measurement emerged as a valuable tool for distinguishing papilloma lesions from CRS. According to the diagnostic protocol based on SCCA-1 and SCCA-2 measurements shown in Figure 3B, the sensitivity was 0.65, specificity was 0.98, PPV was 0.92, and NPV was 0.91 for differentiating IP and other types of papilloma from other diseases.

### 3.5. Perioperative Changes in SCCA-1 and SCCA-2

Postoperative SCCA-1 levels were lower than preoperative SCCA-1 levels in patients with IP and CRS (paired-sample Wilcoxon signed-rank test, *p* < 0.001 and 0.001, respectively). Conversely, SCCA-2 levels did not change in these patients after surgery (paired-sample Wilcoxon signed-rank test, *p* = 0.650 and 0.321, respectively). The postoperative SCCA-2/SCCA-1 ratio (mean ± SD) in the patients with IP was 0.87 ± 0.43, while that in the patients with CRS was 0.74 ± 0.45. There was no significant difference in postoperative SCCA-2/SCCA-1 between IP and CRS.

### 3.6. Influence of Comorbidities and Lifestyle Factors (Smoking and Alcohol Consumption) on SCCA Values

Comorbidities such as lung and skin diseases were investigated for their potential relationship with SCCA levels (Table S3, Supplementary Materials). Of eighteen patients with IP, four (22.2%) had lung disease (one had asthma and three had chronic obstructive pulmonary disease [COPD]), while twenty-two (28.6%) of the seventy-seven patients with CRS had current lung disease (twenty had asthma and two had COPD). Eight patients in the CRS group had a previous history of pneumonia (six cases) or pneumothorax (two cases). There were no significant differences in preoperative SCCA-1 and SCCA-2 levels in the patients with CRS according to current lung disease status (*p* = 0.282 and 0.139, respectively, Mann–Whitney U test). Similarly, in the patients with IP, there were no significant differences in preoperative SCCA-1 and SCCA-2 levels according to current lung disease status (*p* = 0.277 and 0.505, respectively, Mann–Whitney U test). There was only one patient (CRS) with skin disease (chronic polymorphic prurigo).

The results for SCCA-1 and SCCA-2 according to lifestyle factors are also shown in Table S3 (Supplementary Materials). Compared to patients with CRS, those with IP tended to be current or ex-smokers (*p* = 0.07, chi-square test) and habitually consume alcohol (*p* = 0.04, Fisher’s exact test). Preoperative SCCA-1 and SCCA-2 levels were significantly higher in ex-/current smokers with IP than in never smokers with IP (4.8 and 1.3 ng/mL vs. 1.8 and 0.5 ng/mL, respectively) (*p* = 0.035 and 0.035, respectively, Mann–Whitney U test). In contrast, there were no significant differences in SCCA-1 and SCCA-2 levels between never and ex-/current smokers with CRS (*p* = 0.347 and 0.859, respectively, Mann–Whitney U test). There was only one never drinker in the IP group. There was no significant difference in preoperative SCCA-1 and SCCA-2 according to drinking habit in the CRS group (*p* = 0.211 and 0.898, respectively, Mann–Whitney U test).

## 4. Discussion

The results of this study highlight an apparent discrepancy in SCCA-1 levels between patients with IP and those with CRS. Notably, SCCA-1 shows robust diagnostic accuracy in discriminating papilloma (IP and other types of papilloma) from other endoscopically treated sinonasal diseases, as evidenced by ROC analysis. While SCCA is produced by two homologous genes, *SCCA-1* and *SCCA-2*, SCCA-1 plays a pivotal role in the diagnosis of IP, with SCCA-2 providing limited diagnostic utility. Previous analysis of SCCA-2 in CRS, IP, and sinonasal SCC demonstrated that SCCA-2 levels are significantly higher in sinonasal SCC than in CRS and IP when measured using different in-house assay systems [27]. Our results for SCCA-2 levels in IP and CRS were consistent with that report [27]. Considering that the Architect assay can measure SCCA-1 without interference from SCCA-2, previously reported data regarding SCCA in IP [16,20] may predominantly reflect SCCA-1.

Notably, SCCA-1 levels showed a postoperative decline in both IP and CRS patients, which is consistent with previous retrospective studies [15,16,17,18,19,20,27], whereas SCCA-2 levels remain relatively stable in those patients postoperatively. These findings underscore the critical importance of SCCA-1 measurement in the preoperative diagnosis of IP. Although SCCA-2 measurement alone was not effective for the detection of IP, the specificity of SCCA-1 and -2 measurements in combination proved valuable for discriminating papilloma (IP and other types of papilloma) from other sinonasal diseases.

The significance of SCCA-2 measurement in the preoperative detection of IP became apparent in patients with SCCA-1 of ≥1.8 to <3.4 ng/mL. As shown in Figure 2A, SCCA-1 levels positively correlate with SCCA-2 levels in patients with sinonasal diseases. Whereas SCCA-2 levels tended to be higher in IP than in CRS, SCCA-2 levels were lower in IP than in CRS in cases with SCCA-1 of ≥1.8 to <3.4 ng/mL. The IP diagnostic protocol in Figure 3B has the potential for high specificity, PPV, and NPV in IP detection. Importantly, this is the first report on the significance of SCCA-1 and SCCA-2 in the diagnosis of IP based on a prospective study.

The SCCA-2/SCCA-1 ratio was notably lower in patients with IP than in those with other diseases, further demonstrating its potential as a diagnostic marker for IP. In ROC analysis, the AUC of SCCA-2/SCCA-1 for distinguishing papilloma (IP and other types of papilloma) from other diseases was 0.747 (Figure 2D). The ROC analysis results indicate that SCCA-1 outperforms SCCA-2/SCCA-1 as a biomarker for identifying IP lesions. In line with these results, it is important to emphasize that SCCA-1 levels decreased postoperatively while SCCA-2 levels remained relatively unchanged. Among the eighteen patients with IP in this study, only one exhibited elevated SCCA-1 levels after surgery, which was associated with bilateral IP and residual lesions. After removing the residual lesion, the SCCA-1 levels decreased to below 1.8. These findings underscore the potential role of postoperative SCCA-1 levels in ensuring the complete resection of IP. However, it is worth noting that the influence of tumor size and location on SCCA-1 values remains unexplained despite previous studies suggesting a correlation between SCCA-1 and tumor size. Therefore, further investigations are warranted to validate this assumption.

CRS patients with elevated SCCA levels tend to have a history of smoking or mild pulmonary dysfunction [20], and SCCA-1 and SCCA-2 have reported markers of atopic dermatitis [22]. Thus, this study examined the presence of skin and lung diseases as comorbidities and assessed the smoking status of the patients. Neither SCCA-1 nor SCCA-2 was significantly correlated with lung diseases in IP and CRS patients, while the association between skin disease and SCCA could not be evaluated because only one patient had skin disease. Notably, among patients with IP, SCCA-1 and SCCA-2 levels were higher in ex-/current smokers than in never smokers, whereas these associations were not observed in CRS patients. These findings suggest that smoking may influence SCCA levels in IP patients. Given the limited sample size and the unknown mechanisms underlying SCCA elevation, further research is needed to corroborate these findings. In the present study, the cutoff value for SCCA-1 to discriminate IP was determined to be 1.8 ng/mL. Notably, this cutoff was different from the value of 1.5 ng/mL used in previous studies based on the manufacturer’s recommendation. The fluctuation of SCCA levels during long observation periods is still not fully understood [20], partly due to smoking status and comorbidities. Thus, a future study with a larger patient cohort is necessary to establish a more precise optimal cutoff value for IP.

Sinonasal IP is a benign neoplasm with distinctive clinical features, including a high propensity for recurrence and the potential for malignant transformation. Significantly, the surgical approach for treating CRS differs substantially from that for treating IP, with CRS surgery preserving most sinus mucosa. Clinically, preoperative biopsy and imaging assessments are critical in distinguishing IP from various sinus lesions. Specific features of magnetic resonance imaging, such as the gyrus-like appearance on T2-weighted images, and computed tomography findings, such as bony thickening, are significant findings in the diagnosis of IP [28]. However, since IP frequently originates in the middle meatus, it may induce chronic inflammation by obstructing the sinus ostium, potentially leading to the omission of pathological examination in CRS surgery [29]. This study highlights the potential of SCCA-1 and SCCA-2 as useful biomarkers for distinguishing IP from other sinonasal diseases, particularly CRS. Including SCCA-1 and SCCA-2 measurements alongside pathological and imaging evaluations would be beneficial for enhancing the preoperative detection of IP.

Some limitations of this study must be acknowledged, including its relatively small sample size and single-center design. Future research involving larger patient cohorts from diverse populations is needed to validate our findings and establish optimal diagnostic thresholds. Furthermore, the underlying mechanisms of SCCA regulation in sinonasal diseases and its association with lifestyle factors warrant further investigation.

## 5. Conclusions

This study revealed distinct differences in SCCA-1 levels between patients with IP and CRS. SCCA-1 was shown to be a reliable diagnostic marker, outperforming SCCA-2 for discriminating papilloma (IP and other types of papilloma) from other sinonasal diseases and showcasing its value in the preoperative diagnosis of IP. SCCA-1 combined with SCCA-2 measurement provided high specificity, PPV, and NPV in detecting papilloma (IP and other types of papilloma). The postoperative decrease in SCCA-1 levels further underscores its significance, with potential utility in evaluating the completeness of IP resection. While our study did not establish a strong link between SCCA and skin or lung diseases, smoking may influence SCCA levels in patients with IP, and this warrants further investigation. We recommend a cutoff value of 1.8 ng/mL for SCCA-1 in the diagnosis of IP, which is different from the manufacturer’s recommendation. Overall, SCCA-1 and SCCA-2, when combined with traditional diagnostic methods, such as imaging and pathology, hold promise for enhancing the preoperative detection of IP, which would be a valuable contribution to clinical practice.

## Figures and Tables

**Table 1:** Pre- and postoperative SCCA-1 values and the preoperative SCCA-2/SCCA-1 ratio.

	Preop. SCCA-1 (n)	Preop. SCCA-1 (ng/mL)	SD	Postop. SCCA-1 (n)	Postop. SCCA-1 (ng/mL)	SD	Preop. SCCA-2/SCCA-1 (n)	Preop. SCCA-2/SCCA-1	SD
IP	18	4.3	3.0	18	1.3	1.1	18	0.3	0.2
Other types of papilloma	2	2.8	2.2	2	1.2	0.2	2	0.4	0.3
CRS	77	1.2	0.6	75	1.0	0.4	70	0.6	0.3
Hemangioma	1	0.9	NA	1	1.1	NA	1	0.8	NA
Sinonasal cancer	4	1.1	0.5	3	1.4	0.7	3	0.7	0.4

CRS, chronic rhinosinusitis; IP, inverted papilloma; n, number; NA, not available; Postop, postoperative; Preop., preoperative; SD, standard deviation.

**Table 2:** Pre- and postoperative SCCA-2 values.

	Patients with Preoperative SCCA-2, n	Preoperative SCCA-2 (ng/mL)	SD	Patients with Postoperative SCCA-2, n	Postoperative SCCA-2 (ng/mL)	SD
IP	18	1.1	0.9	13	1.1	0.6
Other types of papilloma	2	0.7	0.1	1	0.8	NA
CRS	70	0.7	0.5	62	0.8	0.6
Hemangioma	1	0.7	NA	1	1.2	NA
Sinonasal cancer	3	1.0	0.7	2	1.0	0.9

CRS, chronic rhinosinusitis; IP, inverted papilloma; NA, not available; SD, standard deviation.

**Table 3:** Distribution of preoperative SCCA-1 values.

	Patients with Preoperative SCCA-1, n	Preoperative SCCA-1 (ng/mL)		
		<1.8	≥1.8 to <3.4	≥3.4
IP	18	4	4	10
Other types of papilloma	2	1	0	1
CRS	77	67	10	0
Hemangioma	1	1	0	0
Sinonasal cancer	4	4	0	0

CRS, chronic rhinosinusitis; IP, inverted papilloma; NA, not available; SD, standard deviation.

## Data Availability

The datasets generated and/or analyzed during the present study have not been made publicly available. However, data can be made available from the corresponding author upon reasonable request.

## Supplementary Materials

The following supporting information can be downloaded at https://www.mdpi.com/article/10.3390/jcm13092721/s1, Supplementary Methods; Figure S1: SCCA-1 measurements by Architect^®^; Figure S2: SCCA-2 measurements by the SCCA-2 ELISA kit; Table S1: Primers used for the detection of HPV DNA by PCR; Table S2: Primers used for the detection of viral loads and viral integration in real-time PCR; Table S3: Sinonasal diseases according to comorbidities and lifestyle factors.
