# Supplementary material for: Prospective Analysis of Squamous Cell Carcinoma Antigen-1 and -2 for Diagnosing Sinonasal Inverted Papilloma
Source: J Clin Med. 2024 May 6;13(9):2721. doi: 10.3390/jcm13092721 (PMC11084524; doi:10.3390/jcm13092721)
Supplement: Supplementary file 1 [file jcm-13-02721-s001.zip › jcm-2962996-supplementary.pdf]

Supplementary Materials for

Prospective analysis of squamous cell carcinoma antigen-1 and -2 for diagnosing sinonasal inverted papilloma

Contents

Supplementary Methods
Figure S1. SCCA-1 measurements by the Architect®.
Figure S2. SCCA-2 measurements by the SCCA-2 ELISA kit.
Table S1. Patient characteristics.
Table S2. Preoperative SCCA-2 values in patients with SCCA-1 of ≥1.8 to <3.4 ng/ml.
Table S3. Sinonasal diseases according to comorbidities and lifestyle factors.

Supplementary methods for SCCA-1 and -2 measurement

Recombinant human SCCA-1 (catalog number 230-30005) and SCCA-2 (catalog number 230-30014) were purchased from RayBiotech, Inc. (Peachtree Corners, GA). They were expressed in HEK293T cells (Horizon Discovery, Cambridge, United Kingdom), and SCCA-1 and SCCA-2 were purified by immobilized metal ion affinity chromatography.

An automatic chemiluminescence immunoassay (Architect, Abbott Laboratories, Wiesbaden, Germany) and the Alinity SCC reagent (Abbott Laboratories, Wiesbaden, Germany) were used to determine the cross-reactivity of human SCCA-1 and SCCA-2. The above recombinant SCCA-1 and SCCA-2 were prepared at concentrations of 0, 10, 20, 50, 100, and 200 ng/ml. The Architect immunoassay successfully detected the recombinant SCCA-1, and there was a significantly positive correlation between the amount of recombinant human SCCA-1 and the Abbot SCCA antigen level (Pearson correlation test, r = 0.9794, $p < 0.0001$, Spearman's rank correlation coefficient; Figure S1). These measurements were repeated two times using different samples. In contrast, the recombinant human SCCA-2 was not detected by the Architect system at all. Thus, the Architect system can measure human SCCA-1, but does not detect SCCA-2. There was no crossreactivity between SCCA-1 and SCCA-2 in this system.

The SCCA-2 ELISA kit (Shino-Test Cor., Sagamihara, Japan) was employed to detect recombinant human SCCA-1 and SCCA-2. The recombinant SCCA-1 and SCCA-2 were diluted to concentrations of 0, 10, 20, and 50 ng/ml. The SCCA-2 ELISA kit did not detect the recombinant human SCCA-1, but did detect the recombinant human SCCA-2. These measurements were repeated two times using different samples. There was a significantly positive correlation between the amount of recombinant human SCCA-2 and the SCCA-2 level in the Shino-Test (r = 0.9463, $p = 0.0043$, Spearman's rank correlation coefficient; Figure S2). Therefore, this SCCA-2 ELISA kit can detect the SCCA-2 without cross-reactivity to SCCA-1.

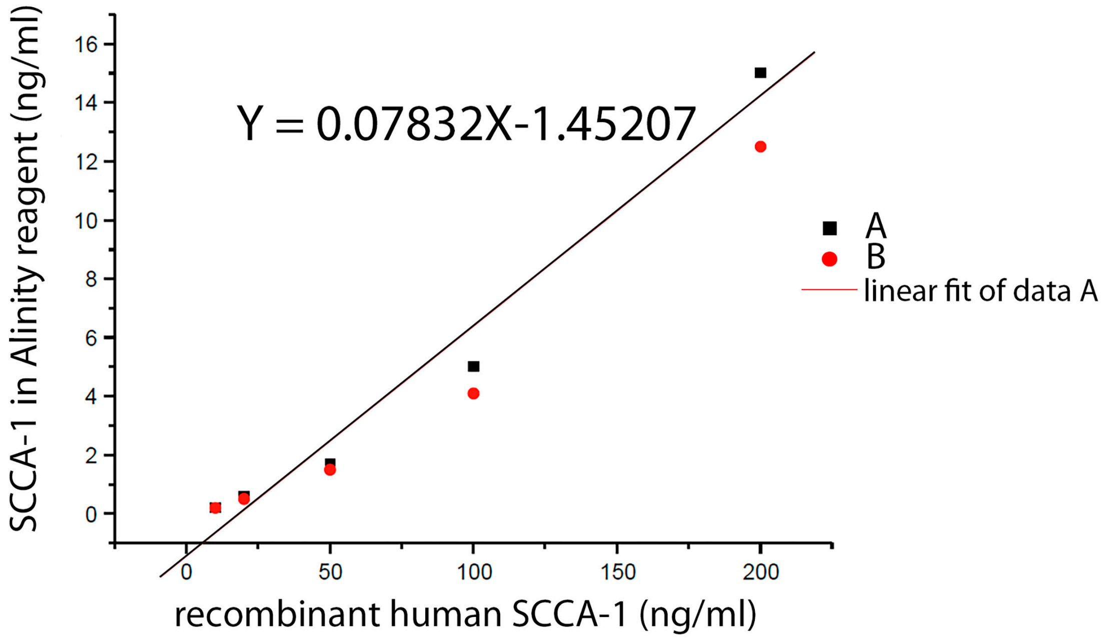

Figure S1. SCCA-1 measurements by the Architect®.

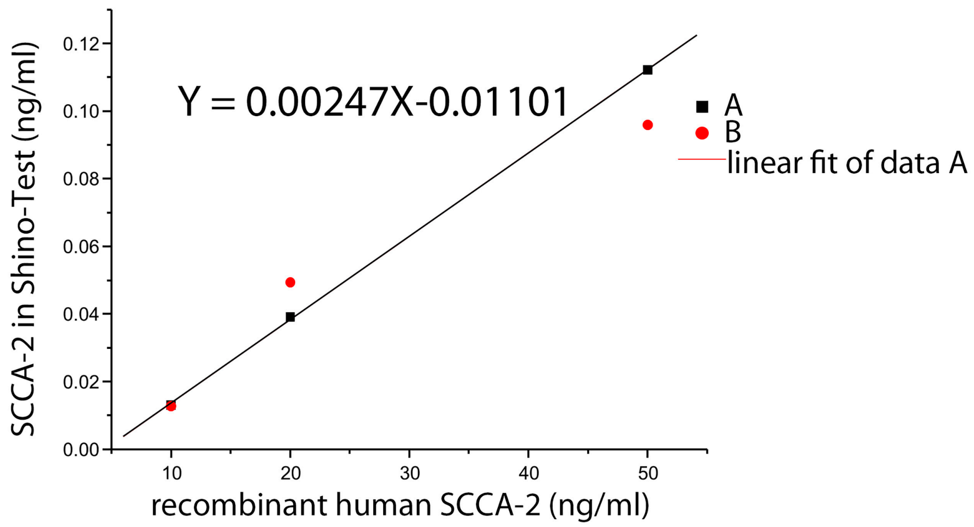

Figure S2. SCCA-2 measurements by the SCCA-2 ELISA kit.

Table S1. Patient characteristics.

		n
Sex		
	male	65
	female	37
Age, years		
	mean 51, median 51	
Affected side		
	unilateral	41
	bilateral	61
Histology		
	inverted papilloma	18
	other types of papilloma	2
	chronic rhinosinusitis	77
	hemangioma	1
	sinonasal carcinoma	4
Smoking habit		
	never	42
	ex-smoker	35
	current smoker	25
Drinking habit		
	never/occasional drinking	26
	habitual drinking	76
Comorbidity		
	asthma	21
	COPD	5
	previous lung disease	8
	skin disease	1

COPD, chronic obstructive pulmonary disease. There was only one case with skin disease (chronic polymorphic prurigo) in this series.

Table S2. Preoperative SCCA-2 values in patients with SCCA-1 of ≥1.8 to <3.4 ng/ml.

	Patients with preoperative SCCA-1of ≥1.8 to <3.4 ng/ml, n	Preoperative SCCA-2 (ng/ml)	
		≤0.6	>0.6
IP	4	2	2
Other types of papilloma	0	0	0
CRS	10	1	9
Hemangioma	0	0	0
Sinonasal cancer	0	0	0

CRS, chronic rhinosinusitis; IP, inverted papilloma.

Table S3. Sinonasal diseases according to comorbidities and lifestyle factors.

	n	Lung diseases		Skin diseases		Smoking habit		Drinking habit	
		none	present	none	present	Never	ex-/current smoker	never/ occasional	habitual
IP	18	14	4	18	0	4	14	1	17
Other types of papilloma	2	2	0	2	0	1	1	1	1
CRS	77	55	22	76	1	35	42	23	54
Hemangioma	1	1	0	1	0	1	0	0	1
Sinonasal caancer	4	4	0	4	0	1	3	1	3

CRS, chronic rhinosinusitis; IP, inverted papilloma.
